# Trend Decomposition for Temperature Compensation in a Radar-Based Structural Health Monitoring System of Wind Turbine Blades

**DOI:** 10.3390/s24030800

**Published:** 2024-01-25

**Authors:** Jonas Simon, Jochen Moll, Viktor Krozer

**Affiliations:** Department of Physics, Goethe University Frankfurt/Main, 60438 Frankfurt, Germany; moll@physik.uni-frankfurt.de (J.M.); krozer@physik.uni-frankfurt.de (V.K.)

**Keywords:** structural health monitoring, damage detection, temperature compensation, seasonal trend decomposition, wind turbine blades, FMCW radar

## Abstract

The compensation of temperature is critical in every structural health monitoring (SHM) system for achieving maximum damage detection performance. This paper analyses a novel approach based on seasonal trend decomposition to eliminate the temperature effect in a radar-based SHM system for wind turbine blades that operates in the frequency band from 58 to 63.5 GHz. While the original seasonal trend decomposition searches for the trend of a periodic signal in its entirety, the new method uses a moving average to determine trends for each point of a periodic signal. The points of the seasonal signal no longer need to have the same trend. Based on the determined trends, the measurement signal can be corrected by temperature effects, providing accurate damage detection results under changing temperature conditions. The performance of the trend decomposition is demonstrated with experimental data obtained during a full-scale fatigue test of a 31 m long wind turbine blade subjected to ambient temperature variations. For comparison, the well-known optimal baseline selection (OBS) approach is used, which is based on multiple baseline measurements at different temperature conditions. The use of metrics, such as the contrast in damage indicators, enables the performance assessment of both methods.

## 1. Introduction

Wind power has become increasingly important as an efficient, renewable energy source in recent decades. The number of wind turbines is increasing from year to year [1]. With more than 17,000 blades now in use worldwide, it is estimated that 3800 are certainly damaged [2]. The common types of damage that can destroy a rotor blade include delamination, debonding, and cracks [3]. This damage usually starts small and grows until the blade collapses. Only close monitoring can detect damage during growth and prevent further damage and costs. The currently applied comprehensive monitoring is carried out by trained industrial climbers. With costs of several thousand dollars per inspection on land and several tens of thousands of dollars offshore, these inspection significantly reduce the profitability of the plants [4]. These circumstances motivate the development of automated, permanent monitoring systems for wind turbines: so-called structural health monitoring systems. The most common and most widely researched systems are based on voltage measurements, acoustic emission, vibration, and ultrasound [5]. Radar-based monitoring approaches offer a number of important advantages in structural monitoring, such as non-contact measurement and the monitoring of large areas. For this reason, the use of radar devices in the monitoring of wind turbines is an increasingly important aspect of research. The approaches to date monitor the rotor blades with sensors from the tower or from the ground, as presented by Nikoubin et al. [6], Zhao and Hong [7], and Mälzer et al. [8].

A new approach integrates the radar sensors directly into the rotor blades with the best possible distribution, determined by simulation. A total of 40 low-cost FMCW radar sensors monitor the entire rotor blade with redundancy and the option for triangulation. Each sensor can monitor the entire environment but only requires a limited field of view. The disadvantages of radar sensors listed by Sun et al. [9] in their overview of monitoring technologies for wind turbines, such as high transmission power and increased power consumption, are thereby relativized. The disadvantage remains in that radar sensors are susceptible to operational and environmental changes. Structural changes, such as damage, change the measured signal. These changes enable an automated system to detect damage. If temperature influences change the signal to a similar extent, damage can go unnoticed or the system can be falsely triggered even though there is no damage.

Temperature influences the measured radar signals through several physical effects. A first, very obvious effect is the temperature-dependent change in the physical properties of the observed environment. The permittivity of the glass fiber reinforced plastics (GFRP) and core materials of the rotor blade, which is primarily important for radar measurements, changes [10,11]. This directly influences the reflection, transmission, and dispersion behavior of electromagnetic waves. Secondly, the temperature has an influence on the electronics of the sensor itself [12,13]. Due to changing conductivities, temperature-dependent changes also occur in the analog-to-digital converter (ADC) and digital-to-analog converter (DAC).

The temperature influence of FMCW radar sensors has also been shown in a variety of works. For example, climate chamber experiments show the influence of temperature and humidity on radar measurements [14]. In the calibration of cloud radars according to Toledo et al. [15], temperature plays a decisive role. These observations strongly imply that an SHM system, which by its very nature should have a high level of accuracy, take any influences into account, and compensate for them if necessary. However, the influence of temperature and other environmental influences has not yet been addressed in radar-based monitoring approaches for wind turbines.

For this reason, this work uses the data from a large-scale experiment to analyze this temperature influence. In a full-scale fatigue test, several damages were caused in a 31 m long rotor blade due to overloading. In addition, temperature fluctuations of several degrees were recorded by each radar sensor during the experiment. The radar data from an intact and a damaged rotor blade at temperature change offer an opportunity to compare the effects of temperature and damage and to evaluate the influence on SHM systems. In order to minimize temperature influences and maximize the sensitivity of radar-based SHM systems, several temperature compensation methods are then considered. A first approach is the seasonal trend decomposition, which determines a trend on the periodic radar signals and can thus eliminate it. Due to the effect of the different temperature influences on the signals, this method proved to be only a slight improvement. A further development significantly improves the compensation of temperature influences. To classify the new method, the results for some selected sensors are compared with the results of the optimal baseline selection (OBS), introduced in ultrasound SHM systems [16], and discussed.

## 2. Materials and Methods

### 2.1. Data Acquisition

**Figure 1:**
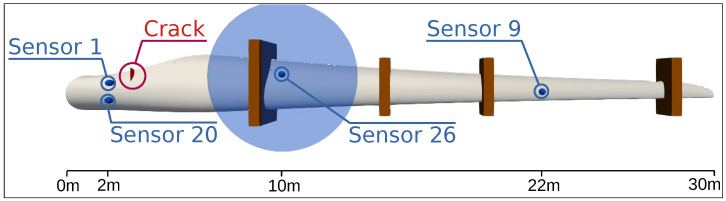
Sketch of the rotor blade with sensor positions, the position of the final crack, and the brown load frames. Sensors 1 and 20 are in direct proximity to the large fatigue crack. Sensors 9 and 26 are too far away to detect the crack, as can be seen from the field of view of sensor 26 (illustrated by the blue circle).

**Figure 2:**
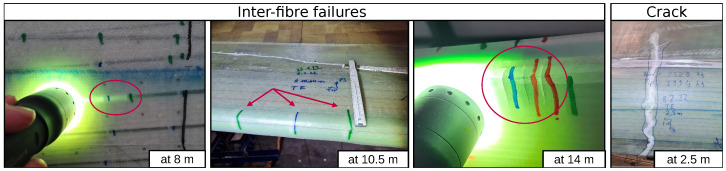
(**left**) Multiple images of fiber damages that become visually detectable with the appropriate light, highlighted by red circles and arrows. (**right**) Image of the crack that ended the fatigue test.

The data were recorded during a full-scale fatigue test. For this purpose, a 31 m long rotor blade in an experimental hall was subjected to a load test according to IEC 61400-23:2014-04 [17]. In incremental steps, the load was increased to such an extent that damage occurred and finally a crack destroyed the blade. A drawing of the blade with some important sensors can be seen in Figure 1. During the experiment, visual inspections were carried out by trained workers and a large number of small damages to the GRP material were documented. In particular, micro-damage in the form of fiber breaks, as shown in Figure 2, occurred across the entire surface. However, the frequency and severity decreases towards the tip, as the sheet was less severely deformed there. This crack can also be seen in Figure 2 on the right. The blade was loaded edge-wise with a piston. Wooden load frames were mounted to provide an optimal load distribution and are illustrated in Figure 1. These load frames have been removed from time to time for vibration analyses and then reinstalled in approximately the same position. Therefore, exact positioning could not be guaranteed and minor structural changes can be expected near any of the load frames.

In order to eliminate any possible influences on the radar signals caused by deformation of the blade, only data from test pauses when the blade was stationary are used in this work. During these test pauses, however, the blade was exposed to environmental influences inside the experimental hall.

A total of 40 radar sensors were placed in the rotor blade. Of these 40 radar sensors, 10 sensors are embedded in the rotor blade material and will not be considered further here, as they behave very individually and completely differently to the glued-on sensors. The embedding in GFRP structures of different thicknesses, depending on the position in the blade, and in particular the unique positioning of glass fiber fabric directly in front of the sensors, makes a holistic analysis impossible. A total of 8 sensors (and a total of 6 glued-on sensors) were damaged during production and were therefore unusable. The low-cost radar sensors used consisted of a BGT60TR13C FMCW 60 GHz radar from Infineon Technologies (Neubiberg, Germany) with a bandwidth of 5.5 GHz, sub-GHz communication, an LIS3DH acceleration sensor including a temperature sensor, and a microcontroller for control.

**Figure 3:**
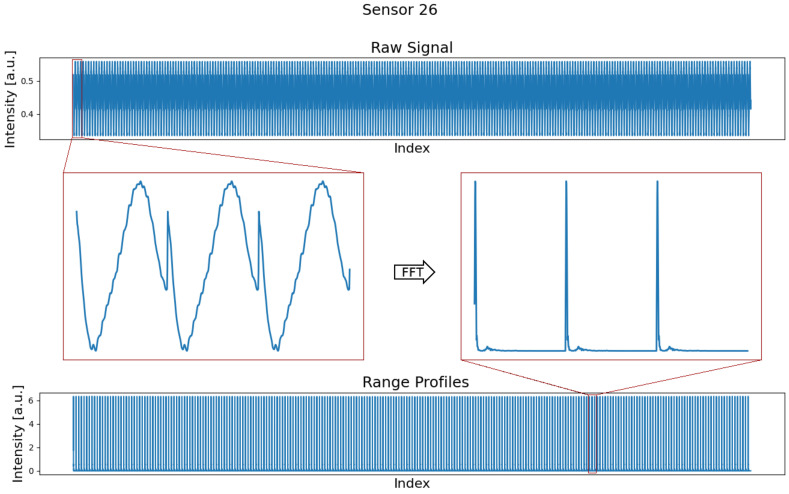
(**top**) Time-series with many concatenated radar signals in the time domain. The zoom window shows three exemplary raw signals corresponding to three frequency ramps. (**bottom**) Representation of many concatenated radar signals after applying the fast Fourier transform (FFT). The zoom window on the right side illustrates three subsequent range profiles corresponding to three frequency ramps after FFT.

The BGT60TRC has 1 transmitting and 3 receiving antennas integrated in a package. The FMCW radar has a spatial resolution of around 2.5 cm and a range of 3.5 m. As is usual with FMCW radar sensors, the transmitted signal is mixed with the emitted signal. By calculating an FFT on the mixed signal, distance-dependent amplitudes, so-called range profiles *s*, can then be generated. The raw measured signal and the calculated range profiles are shown in Figure 3. The sensors are housed in a 3D-printed casing. In order to eliminate any reflections from the housing and near-field artifacts, gating is applied to the range profiles and only amplitudes from 0.2 m are considered.

While the data are transmitted via wireless communication, the sensors are supplied with power via cables. These cables are fixed every few centimeters, but it is conceivable that they can move slightly due to the load on the blade. A more detailed description of the experiment can be found in [18].

### 2.2. Seasonal Trend Decomposition

The seasonal trend decomposition works for measured data $y_{t}$, that is, either the sum

(1) $$y_{t}=S_{t}+T_{t}+R_{t}$$

of a seasonal part $S_{t}$, a trend $T_{t}$ and a residual $R_{t}$, or the quotient

(2) $$y_{t}=S_{t}\cdot T_{t}\cdot R_{t}$$

of these terms [19]. This means that the additive approach assumes that an additive trend lies above the periodic data and the multiplicative approach assumes that a trend scales each data point of the periodic signal. In our problem, the periodic data correspond to the range profiles with 512 data points.

In either case, the trend is then estimated using a moving average

(3) $$T_{t}=\frac{1}{2\cdot k+1}\sum_{j=-(k+1)}^{\left(k+1\right)}y_{t+j}\cdot w_{j}$$

with the order m=2·k+1, where *k* corresponds to the periodicity and is therefore 512. The moving average is based on the common implementation for seasonal trend decomposition with a window in the form

(4) $$w=[\underset{-(k+1)}{0.5}\;\;\underset{-k}{1.0}\;\;\cdots \;\;\underset{-1}{1.0}\;\;\underset{0}{1.0}\;\;\underset{1}{1.0}\;\;\cdots \;\;\underset{k}{1.0}\;\;\underset{k+1}{0.5}.]$$

What is special here is that the points with the same index in the previous and subsequent range profile are only weighted with 0.5.

On the basis of the various influences described (influences on the electronics, temperature-dependent change in permittivity and thus change in reflection and dispersion) and their complex superposition, it is not immediately obvious whether the temperature trend is additive or multiplicative in the case of radar signals. Looking at the phase term [20]

(5) $$Y\left(r,w\right)=exp\left(i\beta t+i\alpha t^{2}\right)\cdot exp\left(i\;{\underline{k}}_{n}\left(T\right)\cdot r_{n}\right),$$

which can be used to calculate the dispersion and signal strength, it can be assumed that temperature changes are multiplicative rather than additive.

A comparative application of the additive and multiplicative method quickly shows that a multiplicative trend reduces the temperature influence while the additive approach does not provide any useful results. From this observation, it can be concluded that a multiplicative approach must be chosen for temperature trends in the case of radar signals. Accordingly, we obtain the adjusted range profiles based on separation of the seasonal part with

(6) $$S_{t}\cdot R_{t}=y_{t}/T_{t}$$

where the residual containing, i.e., noise cannot be separated.

**Figure 4:**
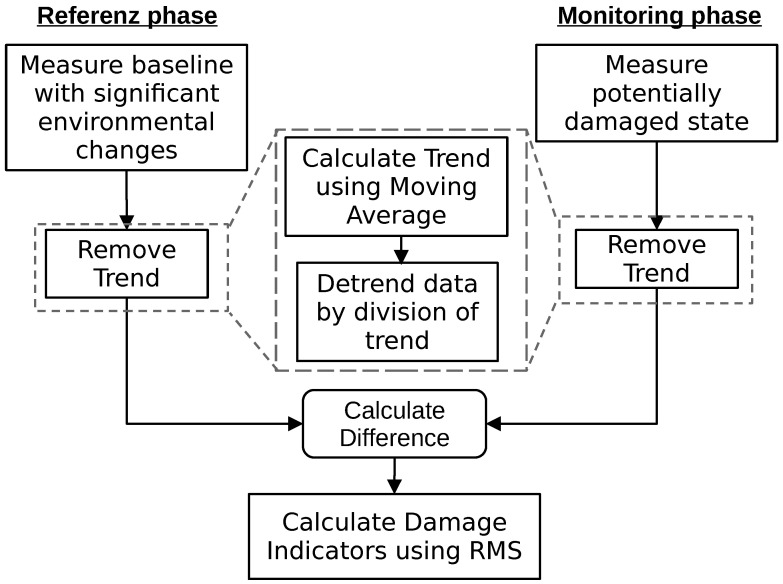
Workflow of processing a measurement for the detection of damage with temperature compensation based on a seasonal trend decomposition.

The procedure with which a structural health monitoring system with the described trend compensation would work is described in Figure 4.

### 2.3. Bin-Specific Trend Compensation

With bin-specific trend compensation (BSTC), a separate trend is determined for each range bin of a series of range profiles. This differs from seasonal trend decomposition, where all points of a range profile share the same trend. This is implemented using slices with a periodicity interval *k* on the original data set. For this purpose, all points except the indices −(*k* + 1), 0 and *k* + 1 are removed from the moving average filter (4). *k* here again corresponds to the length of the given range profiles of 512. The filter becomes

(7) $$w=[\underset{-1}{0.5}\;\;\underset{0}{1.0}\;\;\underset{1}{0.5}]$$

The formula for the trend adapted to a single bin *t* is

(8) $$T_{t}=\frac{1}{2}\sum_{j=-1}^{1}y_{t+j\cdot k}\cdot w_{j}$$

with the periodicity *k*. The formula forms the mean value for the range bin *i* under consideration with the same range bin i−k of the previous measurement and the range bin i+k of the subsequent measurement.

Here, it is no longer possible to simply divide by the trend, as otherwise the ratio between the bins of a range profile would vanish. After dividing by the determined trend, the mean value of each bin would be equal to 1. Therefore, the trend relative to the respective mean value ${\bar{y}}_{t,b}$ of the bin is eliminated. This must be carried out for each bin *b* = 1⋯k. The temperature-independent signal is derived from

(9) $$S_{t,b}\cdot R_{t,b}=y_{t,b}/T_{t,b}\cdot {\bar{y}}_{t,b}$$

A decisive effect that the seasonal trend decomposition has on the seasonal signal $S_{t}$ is that the mean value of the seasonal component $S_{t}$ after adjustment is always the same and independent of the size of $T_{t}$. For this reason, each range profile is also divided by the mean value in bin-specific trend compensation after adjustment of the trends.

While the adjustment of the trend works differently, the workflow is the same as in Figure 4.

### 2.4. Optimal Baseline Selection

For the optimal baseline selection, a number of $N_{T}$ baselines $B_{T_{b}}$ with equidistant temperature steps $\Delta_{T}$ is created. A simple way to obtain these baselines is to extract them from a continuous measurement by averaging the measurements within a $\Delta_{t}$ sized bin. For a measurement at temperature *T*, the baseline $B_{T_{b}}$ is used for evaluation, where $T_{b}-T$ is minimal.

**Figure 5:**
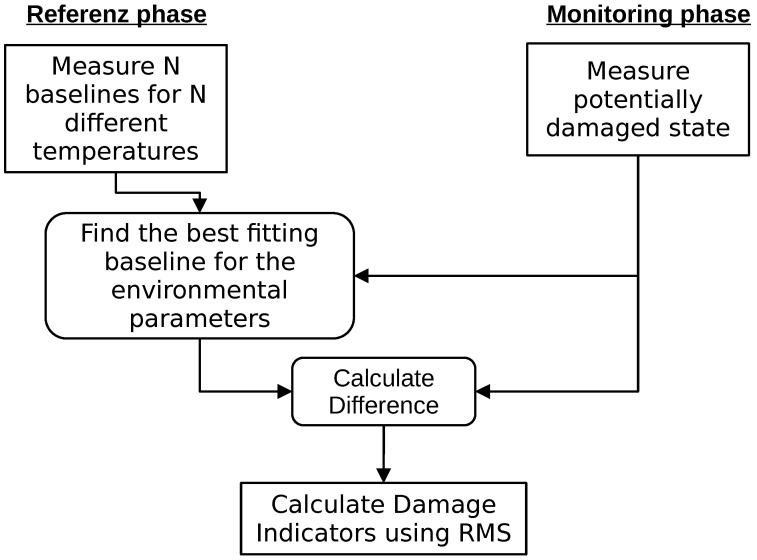
Workflow for application of an optimal baseline selection in structural health monitoring to compensate temperature influences.

The procedure used by a structural health monitoring system with optimal baseline selection is described in Figure 5.

### 2.5. Thresholds

To estimate structural damage based on measured data, a threshold value is usually used for adjustment. If the measurement data exceed the threshold value, the monitoring system assumes a material failure and reacts. The determination of a suitable threshold value is not trivial. A common approach is to calculate the 99th percentile from a set of reference data as the threshold. However, with the 99th percentile, measurements were already above the threshold during the reference period. Outliers in the measurement data may again exceed the threshold. Therefore, one usually requires that several measurements in a row exceed the threshold. As we will see later, the maximum values of our radar measurement are adjacent to each other due to temperature dependence, so this mechanism alone may not be sufficient to prevent false alarms. To ensure that no measurement in the reference condition would trigger an alarm, we add the standard deviation to the 99th percentile and require that this threshold is exceeded five times. In this definition of the threshold, the standard deviation has the advantage that the threshold is correspondingly higher in the case of strongly fluctuating values. The threshold value based on a set of damage indicators DI is thus calculated with

(10) $$Threshold=DI_{0,99}+std\left(DI\right).$$

From the radar data as range profiles, which consist of a set of distance-dependent amplitudes, single values must be made for the comparison with a threshold value. For this purpose, the root mean square (RMS)

(11) $$DI=\sqrt{\sum_{i=0}^{N}{\left(s_{i}-b_{i}\right)}^{2}}$$

of the differences of signal *s* and baseline *b* is calculated as damage indicator DI.

## 3. Results

### 3.1. Temperature Effects

**Figure 6:**
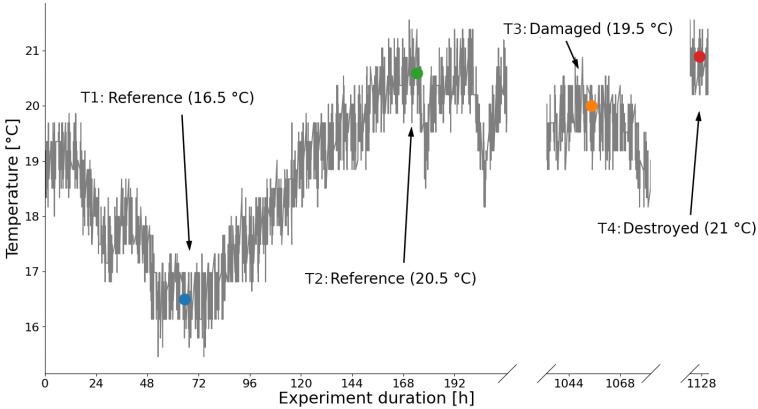
Temperature curve with time points of reference measurement at minimum temperature (blue) and at maximum temperature (green), measurement in damaged state (orange), and measurement in destroyed state (red).

Figure 6 shows the temperature curve of sensor 26 during the experiment. The temperature sensors of the radar systems are not calibrated, so they do not necessarily provide the absolute temperatures, but they do provide the temperature change. The displayed absolute temperatures in Figure 6 are from an external sensor at the blade’s root. The inaccuracies and thus the statistical error of the temperature sensor can be easily recognized from the temperature curve.

To provoke the worst case of temperature influences and prevent the detection of existing damage, the references are compared at minimum and maximum temperature. In the following, the reference measurement at maximum temperature (Reference 20.5 °C) is always used as baseline. The reference measurement at minimum temperature (Reference 16.5 °C) then shows the temperature influence in comparison with the damages and structural changes in later states.

**Figure 7:**
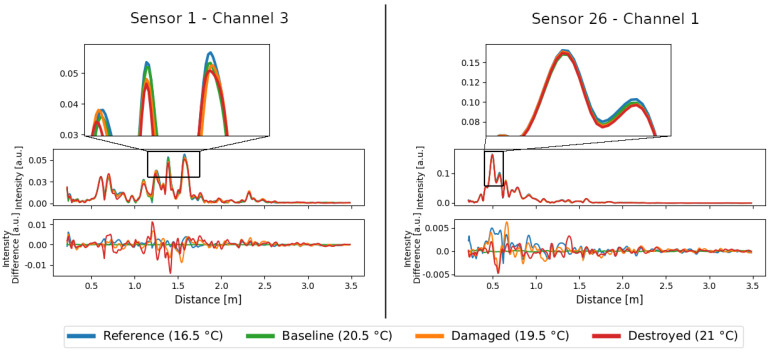
Range profiles of sensor 1 channel 3 on the left and sensor 26 channel 1 on the right. The middle row shows the whole range profiles with a zoom to the peaks of the largest echoes (top row). The bottom row shows the differential range profiles relative to a baseline at 20.5 °C. Temperature effects are visible due to differences to the blue curve.

Damage changes the structure of the rotor blade material [21]. Even minor structural changes alter the reflective behavior of the rotor blade and will result in an amplitude change at the target distance in the corresponding range bin of the range profile [22]. The strength of the change is related to the type and size of the damage and can cause only minor changes relative to the absolute amplitude. In some cases, such as the large crack shown in Figure 1, the amplitude changes are also directly visible in the amplitude plots, as is the case for sensor 1 channel 3 in Figure 7 on the left side at the middle peak of the zoomed in area. It is interesting that the amplitudes of this peak differ significantly from the references, even in the middle of the experiment, although the damage really became visible at the very end of the experiment. This suggests that damage was already present and has grown as a result of the stepwise increase in load. The changes due to temperature, visible by the distance between the blue and green curves, are significantly smaller than the changes due to damage for this peak and thus this single obstacle. For the right amplitude peak in the zoomed in area of sensor 1 channel 3, the temperature effect is significantly higher than any structural changes.

In other cases, such as for sensor 26 channel 1 in Figure 7 on the right side, the changes are much smaller and hardly visible. The changes become visible here only when differences are considered. For this purpose, a baseline at 20.5 °C is subtracted for all states in the bottom plots of Figure 7. Actually, the differences for both reference measurements should be close to zero, since no change has taken place on the blade and no damage can have occurred. However, in the uncompensated difference plot of sensor 26 channel 1 in Figure 7, one can see that the peak of the blue line is strongest at 0.5. This means that the temperature effect here is slightly larger than changes during the experiment. As already mentioned, it can be assumed that the load shears made changes around the rotor blade during the test and small cracks appeared all over the blade, so that minor changes can be expected almost everywhere. The amplitude differences may have increased, but in no case can they have decreased.

These amplitude plots are an excellent way to understand the complexity of the combination of different temperature effects. Some peaks, i.e., echoes, appear to be clearly dependent on the temperature. Other peaks hardly show any dependence on temperature. The reasons for this different behavior are the various orientations, angles, and materials that surfaces have relative to the radar sensor. In addition, there are amplifications of influences due to multiple reflection and scattering, and on top of the temperature changes in the environment, there are the influences of the electronics, which affect every bin. All these effects are combined in a range profile and cannot be separated.

**Figure 8:**
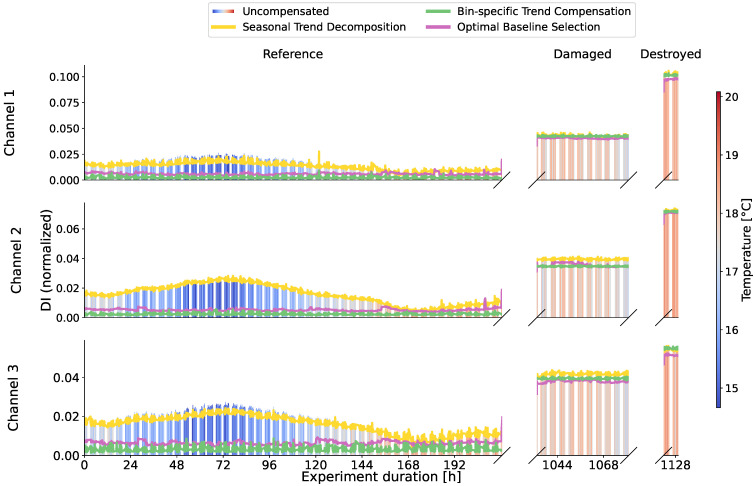
Damage indicators of the uncompensated radar data of sensor 1 as bars with color-coded temperature. The various compensation methods are plotted as lines for direct comparison. Consistently low damage indicators during the reference period mean a low temperature influence.

**Figure 9:**
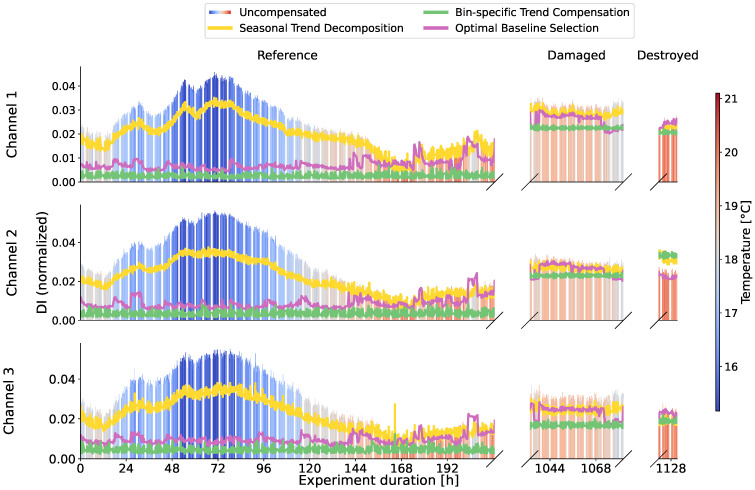
Damage indicators of the uncompensated radar data of sensor 26 as bars with color-coded temperature. The various compensation methods are plotted as lines for direct comparison.

The observations from the amplitudes in Figure 7 are confirmed, because for sensor 1 in Figure 8 damage is detected without any problems even if the temperature is not taken into account. For sensor 26 in Figure 9 the uncompensated damage indicators of the reference phase are in some areas higher than the damage indicators in the potentially damaged and destroyed case. In particular, reference measurements at low temperatures in blue lead to high damage indicators. This is exactly the maximum temperature influence that should be achieved by selecting the baseline at 20.5 °C. Damage indicators from reference measurements at comparable temperatures in red are minimal and are in some cases significantly below the damage indicators in damaged states that have also comparable temperatures. An SHM system should detect damage in the case of sensor 26 but it cannot because of the high values during the reference phase. Sensor 26 describes the problems with environmental influences perfectly and is therefore of great interest.

### 3.2. Seasonal Trend Decomposition

**Figure 10:**
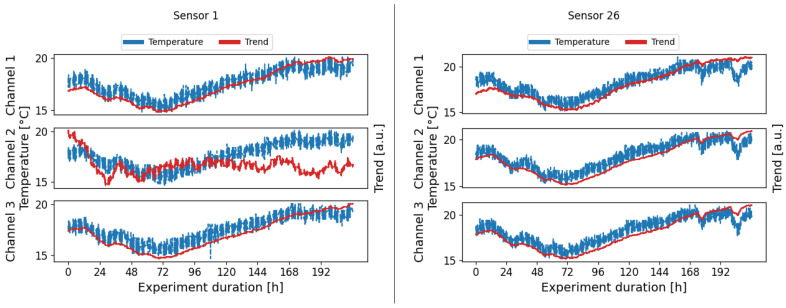
Measured temperature and calculated trends for sensor 1 (**left**) and sensor 26 (**right**). With a few exceptions, the calculated trends show good coverage with the measured temperatures and also include temperature fluctuations due to the heating of the experimental hall.

Using seasonal trend decomposition, an attempt shall now be made to remove the temperature trend from the range profiles. For this, a moving average is applied to the range profiles. Figure 10 shows the measured temperatures and the trends resulting from the moving average. In most cases, the trends agree very well with the measured signals. It should be mentioned here that all trends are inversely proportional for all sensors and are plotted inverted in Figure 10. For some sensors, individual trends do not match the measured temperature at all, as can be seen for example for sensor 1 channel 2 in Figure 10. Reasons for this could be the unique environment for each sensor, the placement in the housing, and interference with it or manufacturing variations. Accordingly, no temperature trend can be removed with the seasonal trend decomposition, as can be seen in Figure 8 for channel 2, where the uncompensated data do not differ from the trend-adjusted data in yellow. In other channels shown in Figure 8 and Figure 9, where a trend similar to the temperature curve was recognized, the damage indicators reduce in the area of lower temperatures and thus temperatures deviating from the baseline. It can be observed overall that the damage indicators for low temperatures are still higher than for high temperatures and the contour of the uncompensated curve is maintained.

**Figure 11:**
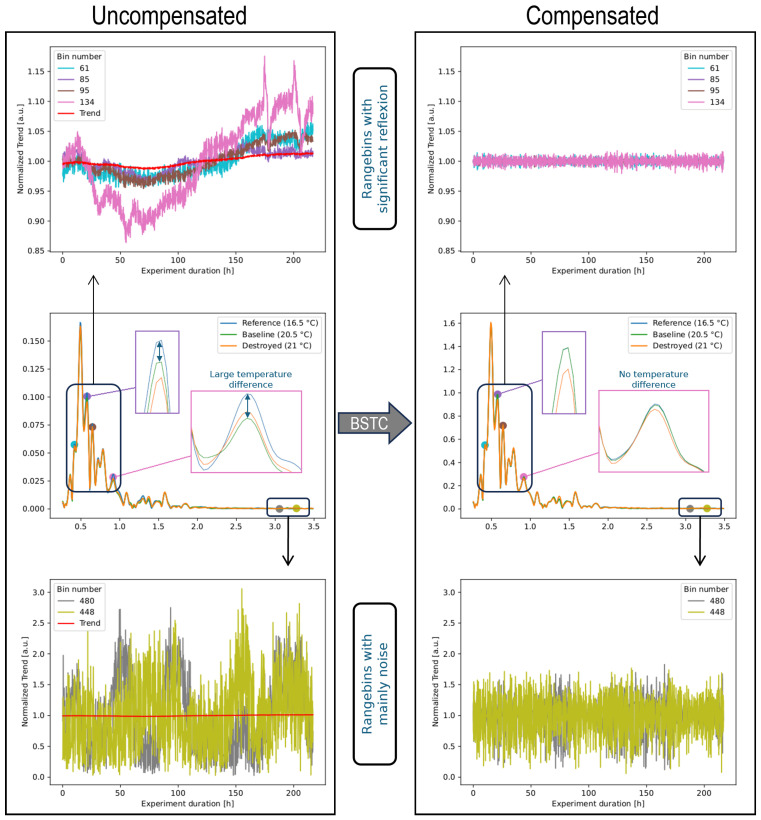
Individual trends for a few selected range bins of sensor 26 channel 3. Not all bins have a temperature dependency and the trends of the bins are generally very different. Bin-specific trend compensation removes these trends and reduces the offset due to temperature in the range profiles—shown by the zoomed areas—significantly.

A trend compensation should equalize the range profiles and lead to constant DIs in each state of the experiment. Figure 11 shows the individual trends for some selected range bins. On the left column, it becomes clear that the range bins show completely different trends. While most bins in the areas with clear reflections, i.e., amplitudes greater than 0, show actual trends and some are roughly similar to the trend in the seasonal trend decomposition or its reversal, other bins like bin 33 deviate from this. In the areas without significant reflections, no trends can be recognized that can be linked to the temperature. Compensation with a global trend, as is the case with the seasonal trend decomposition, cannot eliminate temperature influences on range bins that change individually.

### 3.3. Bin-Specific Trend Compensation

The bin-specific trend compensation compensates every bin of the range profile individually to address this problem. The results of this are plotted as a bin-specific trend compensation in Figure 8 and Figure 9. After compensation, the data are no longer influenced by high or low temperatures. The damage indicators are almost constant and only show measurement inaccuracies.

The effect on individual bins and peaks is illustrated in Figure 11. There, the changes caused by the BSTC are shown in the right-hand column in comparison to the uncompensated data in the left-hand column. The individual trends in the top and bottom row disappear due to the compensation. The range profiles in the same, undamaged state move closer together, as can be seen from the blue and green curves in the zoomed areas.

### 3.4. Optimal Baseline Selection

The results of the optimal baseline selection are used for comparison. For this purpose, the measurements in the reference state with a $\Delta_{T}$ of 1.0 °C were grouped and averaged. The $\Delta_{T}$ of 1.0 °C was chosen in order to use the smallest possible value, but a further reduction was not considered useful due to the strong fluctuations of the temperature sensors as shown in Figure 6. The result is shown in Figure 8 and Figure 9 as a pink curve.

**Figure 12:**
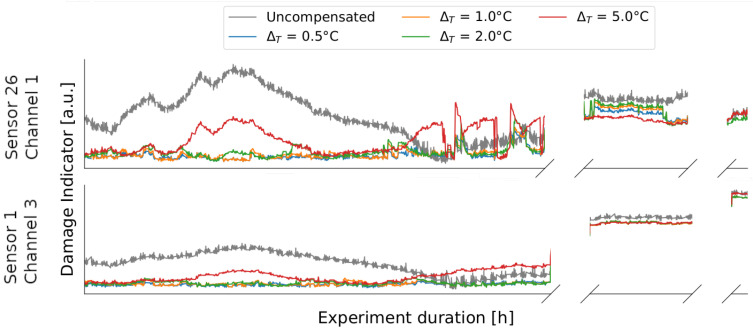
Damage indicator curves of sensor 26 channel 1 and sensor 1 channel 3 for different $\Delta_{T}$ of the OBS method compared to the uncompensated data. As $\Delta_{T}$ decreases, temperature influences increasingly disappear.

To obtain an impression of the influence of the size of the $\Delta_{T}$, the results for different bin sizes are plotted in Figure 12 for channel 3 of sensor 1 and channel 1 of sensor 26. While quite clear temperature influences are still visible for $\Delta_{T}=5.0$ °C and larger jumps become apparent when changing from one baseline to another, the jumps also become smaller as $\Delta_{T}$ becomes smaller and the damage indicators increasingly lose temperature dependence. Between $\Delta_{T}=0.5$ °C and $\Delta_{T}=1.0$ °C, hardly any improvement can be seen. Both sensors behave identically with regard to the $\Delta_{T}$. This overall behavior agrees well with OBS in other application areas such as guided ultrasound waves [23].

**Figure 13:**
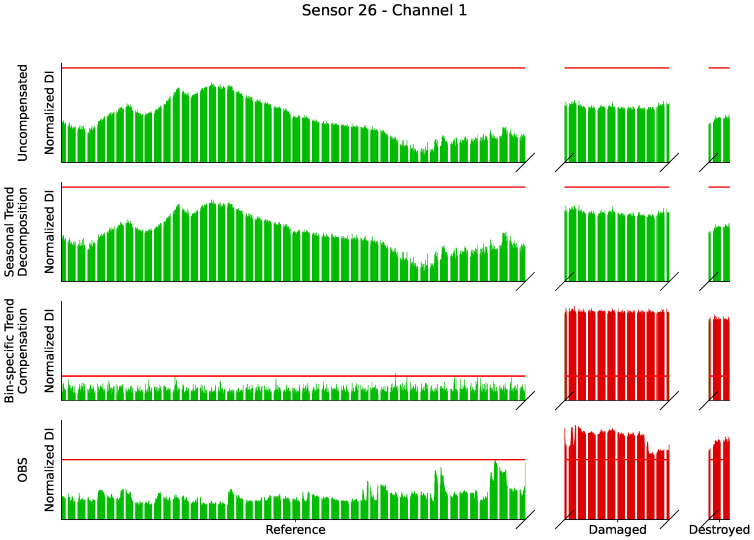
Damage indicators for sensor 26 channel 1 for uncompensated (top) and the 3 compensation methods in red if they would trigger the damage detection system according to the calculated thresholds and in green if not. Only bin-specific trend compensation and optimal baseline selection with $\Delta_{T}$ of 1.0 °C would detect damages.

Figure 13 shows the results of a damage detection system for sensor 26 channel 1 based on the threshold values from Section 2.5 and divided into the uncompensated and compensated cases. Green values are below the threshold value or too few measurements in succession exceed the threshold value. Red bars with at least 5 consecutive values above the threshold value would trigger the damage detection and report damage. Without temperature compensation, no damage would have been detected for this channel. The trend adjustment based on the seasonal trend decomposition means that the damage indicators in the damaged case are less far below the threshold value; damage would still not have been detected. The DIs from the BSTC are well above the threshold value in the damaged and destroyed state and an SHM system would report damage. Similarly, the DIs calculated with OBS in the reference state show a clear difference to the damaged DIs and damage would have been detected.

**Figure 14:**
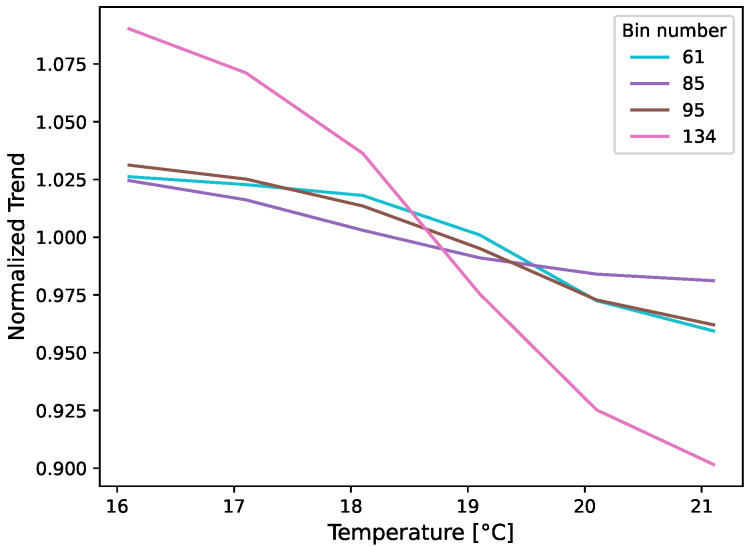
Trends of selected bins with special echoes shown in Figure 11 as a function of temperature. The curves are individual and non-linear.

In addition to the trend curves from Figure 11, in which the trends are plotted over time, the baselines of the OBS method can be used to plot the trends as a function of temperature. This is performed in Figure 14. On the one hand, it is noticeable that the bins are influenced to varying degrees by the temperature, but it should also be noted that existing dependencies appear to be non-linear. This is consistent with previous observations with other radar sensors [14].

### 3.5. Sensor Overview

**Figure 15:**
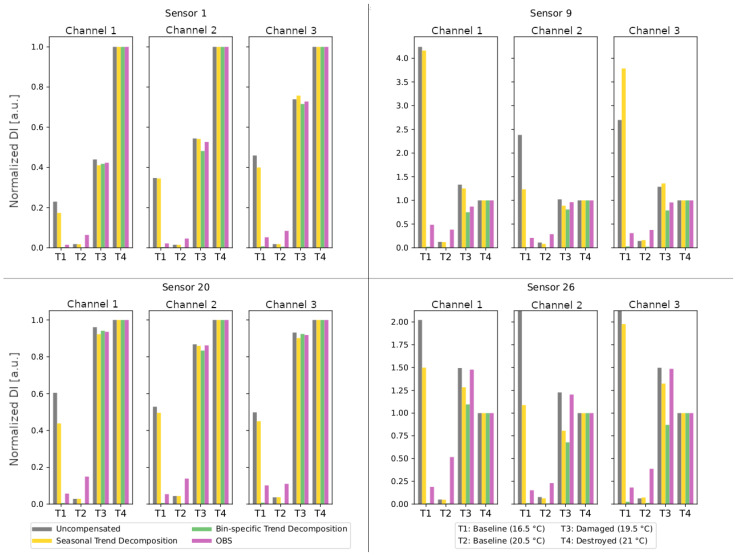
Comparison of damage indicators without compensation (gray), with seasonal trend decomposition (yellow), with bin-specific trend compensation (green) and compensation with optimal baseline selection (pink) for all channels of sensors 1, 9, 20 and 26.

Finally, damage indicators of all three channels are summarized for several sensors from different blade areas in Figure 15. For easier comparison, the DIs are normalized to the value in the destroyed state. The sensor positions can be found in Figure 1. Sensors 1 and 20 are in close proximity to the crack that led to the end of the blade test. Sensor 26 is in the direct vicinity of one of the temporarily removed load frames. Sensor 9 is located in the area of the blade tip and in the middle between two load frames. Hardly any change is visible here, even with OBS. While the damage indicators of the uncompensated data clearly show damage for sensors close to the crack, compensation is necessary for sensor 26. All sensors show a similar effect on damage indicators due to the compensation procedures: The uncompensated data have a high damage indicator for the reference measurement at 16.5 °C (T1) due to the provoked maximum temperature effect and a very low damage indicator for the reference measurement at 20.5 °C (T2), which is itself part of the baseline. The compensation based on the trend by the seasonal trend decomposition leads to slightly lower damage indicators at T1 but also an increase at T2. The optimal baseline selection further reduces the damage indicator at T1, while the damage indicator at T2 increases less strongly. The bin-specific trend compensation performs great and eliminates the DIs at T1 and T2 almost completely.

## 4. Discussion

The data from the fatigue test showed that temperature influences the radar signals significantly. This can even lead to temperature effects being greater than the signal change due to structural changes and damage. In addition, the temperature here only changed by around 6 °C, whereas temperatures at a wind turbine in the field can change by more than 10 °C over the course of the day and by more than 30 °C between winter and summer. Temperature compensation increases the sensitivity of an SHM system and is therefore absolutely essential. For sensors close to the large crack, such as sensor 1 and sensor 20, compensation is not absolutely necessary to detect damage. However, in the case of sensor 26, which is shown here in great detail, the many documented small damages only become visible when the temperature of the crack is higher than the temperature of the crack.

A detailed analysis of the range profiles in the Figure 7 and Figure 11 shows that each range bin has a very individual temperature dependence. Motivated by this, the adaptation of the seasonal trend decomposition results in a new method that determines a corresponding trend for each bin. This so-called bin-specific trend compensation (BSTC) shows much better results than looking at the range profiles as seasonal data. The reason for this is that the range profiles do not have a global trend, but each range bin has its own trend. The reason for this in turn lies in the various temperature-dependent effects, which combine to varying degrees in the bins. A bin that does not contain any significant reflections is only affected by influences on the electronics. A bin that contains the reflections of several objects or surfaces is affected by a complex combination of influences on the electronics, the reflections and transmissions, and multiscattering.

The BSTC also shows very good results in comparison to optimal baseline selection. The realization that each bin has its own trend, which is determined by the environment and the echoes, explains why also OBS works so well for radar signals. The baselines at equidistant temperatures provide a discrete function for each bin. This allows to compensate the temperature-dependent behavior well, as long as there is a baseline close to the current temperature.

By dividing the trend, the level of data is adjusted in the seasonal trend decomposition. This is no longer the case with bin-specific analysis, where the bins are adjusted relative to their mean value, as otherwise the relationship between the bins and thus the amplitudes would be lost. As long as the states or measurements to be compared take place in comparable temperature ranges or more precisely, as long as the mean values of measurement series are similar, the trend compensation works excellently. The BSTC was divided by the mean value in order to equalize the absolute values at different ambient values. Since the temperature dependence from Figure 14 has shown that the trends are not linear and deviate in their gradient, this division by the mean value only works on average. For most bins, the trend of the mean value will not match the trend of the bin and the absolute values of the bins at BSTC will change if measurement series are recorded at different temperature intervals. The mean values are similar enough for the comparison of the experiment considered here. This is also clearly shown in Figure 8 and Figure 9, where the DIs of OBS and BSTC are at a comparable level. The correction of trends seems very suitable for comparing the data in different test states to recognize changes. Nevertheless, an investigation of the comparability for non-matching temperature intervals would be very interesting in the future, preferably based on measurements over larger temperature ranges.

The fluctuations in the DI are smaller than in the OBS. By eliminating the trend, there are also no jumps or strong changes in the course of the BSTC compensated data. The remaining fluctuations in the BSTC are due to measurement inaccuracies. The DIs in the reference period for OBS, some of which are larger in direct comparison, are due to the differences between the temperature and the baseline. The OBS method stores values individually for each bin in discrete temperature steps and therefore provides a rough function for eliminating trends. The BSTC, on the other hand, generates a gapless, continuous curve for compensation.

If we look at the trend progression in Figure 10, which at least represents the trend on average for the range profile, it is noticeable that the trend corresponds well with the temperature in most areas. Towards the end, the trend continues to rise, although the temperature has stagnated. This indicates that any other environmental influence or another effect has added up, which was not covered by the recording of the temperatures. With the OBS method used, only the temperature is taken into account. This also explains why the damage indicators for OBS slightly increase at the end of the reference period in Figure 9 and may also form a poor baseline for the measurements in the destroyed state at maximum temperatures. On the other hand, seasonal trend decomposition and BSTC should eliminate trends due to any and all environmental influences.

These differences explain the better results shown here for BSTC compared to the established and SHM system-independent OBS method. A final advantage of BSTC is a significantly reduced memory requirement, as a single range profile has to be saved, whereas with OBS the number of reference values can be significantly higher depending on the step sizes and the number of influencing variables. This can be particularly advantageous for modern sensor networks with evaluation on the sensor node.

## 5. Conclusions

This work demonstrated that temperature variations during a full-scale fatigue test of a wind turbine blade have a non-negligible influence on radar signals. These temperature influences can also obscure damage even for small temperature changes of only a few degrees Celsius. This clearly shows that temperature compensation is necessary in a radar-based SHM system if the monitored structure is exposed to temperature changes.

With the bin-specific trend compensation and optimal baseline selection presented here, damage can be detected that would otherwise remain hidden due to temperature-dependent amplitude changes. Since they are independent of the specific temperature effects, these approaches are transferable to other radar sensors and application scenarios. The results motivate a more extensive and detailed investigation in the future. How the range profiles change when the temperature changes by more than 10 °C daily or 30 °C over the year cannot be estimated from the data analyzed here. It would also be interesting to see how methods perform in direct comparison with larger temperature intervals.

## Data Availability

The datasets presented in this article are not readily available because of their size and storage space requirements. Requests to access the datasets or preprocessed parts should be directed to simon@physik.uni-frankfurt.de.

## Abbreviations

The following abbreviations are used in this manuscript:

SHM	Structural Health Monitoring
FMCW	Frequency Modulated Continuous Wave
GFRP	Glass fiber reinforced plastics
OBS	Optimal Baseline Selection
BSTC	Bin-specific Trend Compensation
RMS	Root Mean Square
DI	Damage Indicator

## References

[1] Bošnjaković M., Katinić M., Santa R., Marić D. (2022). Wind Turbine Technology Trends. Appl. Sci..

[2] Chen X. (2018). Fracture of wind turbine blades in operation-Part I: A comprehensive forensic investigation. Wind Energy.

[3] Soerensen B.F., Joergensen E., Debel C.P., Jensen F.M., Jensen H.M., Jacobsen T., Halling K.M. (2004). Improved Design of Large Wind Turbine Blade of Fibre Composites Based on Studies of Scale Effects (Phase 1)-Summary Report.

[4] Wymore M.L., Van Dam J.E., Ceylan H., Qiao D. (2015). A survey of health monitoring systems for wind turbines. Renew. Sustain. Energy Rev..

[5] Du Y., Zhou S., Jing X., Peng Y., Wu H., Kwok N. (2020). Damage detection techniques for wind turbine blades: A review. Mech. Syst. Signal Process..

[6] Nikoubin T., Muñoz-Ferreras J.-M., Gómez-García R., Liang D., Li C. Structural health monitoring of wind turbines using a low-cost portable k-band radar: An ab-initio field investigation. Proceedings of the IEEE Topical Conference on Wireless Sensors and Sensor Networks (WiSNet).

[7] Zhao H., Chen G., Hong H., Zhu X. (2021). Remote Structural Health Monitoring for Industrial Wind Turbines Using Short-Range Doppler Radar. IEEE Trans. Instrum. Meas..

[8] Mälzer M., Beck S., Alipek S., Reichart E., Moll J., Krozer V., Oikonomopoulos C., Kassner J., Hägelen M., Heinecke T. Radar-based structural monitoring of wind turbines blades: Field results from two operational wind turbines. Proceedings of the 14th International Workshop on Structural Health Monitoring.

[9] Sun S., Wang T., Chu F. (2022). In-situ condition monitoring of wind turbine blades: A critical and systematic review of techniques, challenges, and futures. Renew. Sustain. Energy Rev..

[10] Vassilikou-Dova A., Kalogeras I.M., Menczel J.D., Prime R.B. (2009). Dielectric Analysis (DEA). Thermal Analysis of Polymers.

[11] Ilic J., Buschow K.H.J., Cahn R.W., Flemings M.C., Ilschner B., Kramer E.J., Mahajan S., Veyssière P. (2001). Wood: Electrical Properties. Encyclopedia of Materials: Science and Technology.

[12] Zhong L., Fu G., Lu J. A research for influence of temperature on T/R module in radar. Proceedings of the Prognostics and System Health Management Conference (PHM).

[13] Will C., Mann S., Michler F., Reissland T., Lurz F., Weigel R., Koelpin A. Error compensation of the temperature influence on radar based displacement measurements. Proceedings of the 2017 IEEE Asia Pacific Microwave Conference (APMC).

[14] Simon J., Maetz T., Moll J., Krozer V., Krause S. Experimental results on the influence of temperature and humidity on FMCW radar signals at 60 GHz. Proceedings of the 15th European Conference on Antennas and Propagation (EuCAP).

[15] Toledo F., Delanoë J., Haeffelin M., Dupont J.-C., Jorquera S., Le Gac C. (2020). Absolute calibration method for frequency-modulated continuous wave (FMCW) cloud radars based on corner reflectors. Atmos. Meas. Tech..

[16] Lu Y., Michaels J.E. (2005). A methodology for structural health monitoring with diffuse ultrasonic waves in the presence of temperature variations. Ultrasonics.

[17] (2014). Full-scale structural testing of rotor blades.

[18] Simon J., Kurin T., Moll J., Bagemiel O., Wedel R., Krause S., Lurz F., Nuber A., Issakov V., Krozer V. (2023). Embedded radar networks for damage detection in wind turbine blades: Validation in a full-scale fatigue test. Struct. Health Monit..

[19] Hyndman R.J., Athanasopoulos G. (2018). Forecasting: Principles and Practice.

[20] Soumekh M. (1999). Synthetic Aperture Radar Signal Processing with MATLAB Algorithms.

[21] Safri S.N.A.B., Sultan M.T.H., Jawaid M., Jawaid M., Thariq M., Saba N. (2019). Damage analysis of glass fiber reinforced composites. Durability and Life Prediction in Biocomposites, Fibre-Reinforced Composites and Hybrid Composites.

[22] Moll J., Maetz T., Maelzer M., Krozer V., Mischke K., Krause S., Bagemiel O., Nuber A., Kremling S., Kurin T. (2021). Radar-based monitoring of glass fiber reinforced composites during fatigue testing. Struct. Control. Health Monit..

[23] Gorgin R., Luo Y., Wu Z. (2020). Environmental and operational conditions effects on Lamb wave based structural health monitoring systems: A review. Ultrasonics.

